# Cost-Effectiveness Analysis of Risk Factor-Based Lung Cancer Screening Program by Low-Dose Computer Tomography in Current Smokers in China

**DOI:** 10.3390/cancers15184445

**Published:** 2023-09-06

**Authors:** Tiantian Zhang, Xudong Chen, Caichen Li, Xiaoqin Wen, Tengfei Lin, Jiaxing Huang, Jianxing He, Nanshan Zhong, Jie Jiang, Wenhua Liang

**Affiliations:** 1 College of Pharmacy/Guangdong-Hong Kong-Marco Greater Bay Area (GBA), Institue for Real-World Value and Evidence of Drugs and Medical Devices/Southern Institute of Pharmacoeconomics and Health Technology Assessment/International Cooperative Laboratory of Traditional Chinese Medicine Modernization and Innovative Drug, Development of Ministry of Education (MOE) of China, Jinan University, Guangzhou 510632, China; 2 Guangzhou Huabo Biopharmaceutical Research Institute, Guangzhou 510010, China; 3 Department of Thoracic Surgery and Oncology, the First Affiliated Hospital of Guangzhou Medical University, Guangzhou Institute of Respiratory Health, China State Key Laboratory of Respiratory Disease, National Clinical Research Center for Respiratory Disease, Guangzhou 510120, China; 4 Shenzhen Institutes of Advanced Technology, Chinese Academy of Sciences, Shenzhen 518000, China; 5 Department of Respiratory Medicine, the First Affiliated Hospital of Guangzhou Medical University, Guangzhou Institute of Respiratory Health, China State Key Laboratory of Respiratory Disease, National Clinical Research Center for Respiratory Disease, Guangzhou 510120, China

**Keywords:** lung cancer, LDCT screening, current smokers, cost-effectiveness analysis, China

## Abstract

**Simple Summary:**

Low-dose computed tomography (LDCT) has become the standard approach for lung cancer screening, but the definition of populations at high risk of lung cancer still remains inconsistent. Several studies on the cost-effectiveness analysis of LDCT screening for lung cancer in China were identified. The majority of them have focused on the cost-effectiveness analysis of LDCT screening with different starting ages. This economic evaluation assessed up to 36 LDCT screening strategies for current smokers with different starting ages, stopping ages and smoking eligibility criteria. The findings proved that annual screening for those aged 55 to 79 who smoked more than 20 pack-years could be the most recommended strategy for current smokers in the whole of China. Compared with previous studies in China, we provided a more extensive economic evaluation of LDCT screening and provided references and suggestions for the update of the screening guidelines.

**Abstract:**

Although the effectiveness of lung cancer screening by low-dose computed tomography (LDCT) could be shown in China, there could be variation in the evidence concerning the economic impact. Our study explores the cost-effectiveness of lung cancer screening and optimizes the best definition of a high-risk population. A Markov model consisting of the natural history and post-diagnosis states was constructed to estimate the costs and quality-adjusted life years (QALYs) of LDCT screening compared with no screening. A total of 36 distinct risk factor-based screening strategies were assessed by incorporating starting ages of 40, 45, 50, 55, 60 and 65 years, stopping ages of 69, 74 and 79 years as well as smoking eligibility criteria. Screening data came from community-based mass screening with LDCT for lung cancer in Guangzhou. Compared with no screening, all screening scenarios led to incremental costs and QALYs. When the willingness-to-pay (WTP) threshold was USD37,653, three times the gross domestic product (GDP) per capita in China, six of nine strategies on the efficiency frontier may be cost-effective. Annual screening between 55 and 79 years of age for those who smoked more than 20 pack-years, which yielded an incremental cost-effectiveness ratio (ICER) of USD35,000.00 per QALY gained, was considered optimal. In sensitivity analyses, the result was stable in most cases. The trends of the results are roughly the same in scenario analyses. According to the WTP threshold of different regions, the optimal screening strategies were annual screening for those who smoked more than 20 pack-years, between 50 and 79 years of age in Zhejiang province, 55–79 years in Guangdong province and 65–74 years in Yunnan province. However, annual screening was unlikely to be cost-effective in Heilongjiang province under our modelling assumptions, indicating that tailored screening policies should be made regionally according to the local epidemiological and economic situation.

## 1. Introduction

Lung cancer is the most common form of malignant tumor with the highest incident rate and mortality rate worldwide [1,2,3]. In China, lung cancer is the main cause of cancer death both in males and females [4,5]. Despite the 5-year survival rate for clinical stage I after surgery being nearly 70%, most patients are in advanced stages when clinically diagnosed and miss the chance of surgical operation with an age-standardized 5-year survival rate of only 16.1% to 19.7% [6,7]. Smoking is the major risk factor for lung cancer. The prevalence of current smoking is still at a high level in China, which leads to a significant disease burden of lung cancer [8,9]. Fortunately, the National Lung Screening Trial (NLST) demonstrated a reduction in lung cancer mortality of 20% in high-risk populations who underwent three rounds of annual low-dose computed tomography (LDCT) compared with chest radiography (X-ray) [10]. Even higher cancer detection rates were reported after clinical adoption in the United States and validated implementation into routine practice [11].

LDCT has a better performance in detecting early-stage lung cancer in high-risk populations, which is often defined by age and smoking history. For current smokers, the inclusion criteria of LDCT screening from the National Comprehensive Cancer Network (NCCN) Version 2.2023 was among adults aged 50 years or above with a smoking exposure of 20 pack-years [12]. Lung cancer screening was also recommended for those aged 50 to 80 years who smoked more than 20 pack-years by the American College of Radiology (ACR) and the US Preventive Services Task Force (USPSTF) [13,14]. However, for the differences in the epidemiology of lung cancer worldwide, the criteria of high-risk populations remained distinct. Both the Canadian Task Force on Preventive Health Care and the South African Thoracic Society recommended lung cancer screening for those aged 55 through 74 who smoked at least 30 pack-years [15,16]. In China, the definitions of high-risk individuals vary in different guidelines. According to the well-recognized guidelines presented, LDCT was recommended for those aged 50–74 years with a smoking history of 20 pack-years (2018 China guideline-recommended strategy from the National Health and Family Planning Commission), 50–74 years with a smoking history of 30 pack-years (2021 China guideline-recommended strategy from the National Cancer Center of China) or those older than 45 years who smoke more than 20 pack-years (2022 China guideline-recommended strategy from the Chinese Medical Association) [17,18,19]. It appears that although previous studies have provided clarity on key issues of lung cancer screening, uncertainty still remains about the definition of a high-risk population [20].

According to our previous study involving 117,586 Chinese participants, the proportion of stage I lung cancer was much higher in the population undergoing screening at 40 or 45 years of age than that in the population undergoing screening at 50 or 55 years of age, indicating that the start of screening at 40 or 45 years may bring more survival benefit [21]. Theoretically, loose inclusion criteria to give LDCT wider coverage would bring more clinical and survival benefits by including more participants. However, due to the high cost, high false-positive rate, overdiagnosis and overtreatment brought by LDCT screening as well as the high carcinogenic incidence caused by LDCT radiation, LDCT screening has been controversial [22]. Researchers have been devoted to finding the optimal strategy by conducting a cost-effectiveness analysis of LDCT screening for lung cancer which balanced harms and cost with benefit and outcome in the past decades. A study from the United States and Germany found that starting LDCT screening at 55 years of age may be cost-effective for those with a smoking history of at least 20 pack-years or 20 cigarettes per day who carry a disproportionately high disease burden from lung cancer at the common WTP threshold [23,24]. In America, another study demonstrated that the 2021 USPSTF recommendation was cost-effective [25]. In Asia, lung cancer screening programs were also shown to be cost-effective for heavy smokers aged 55–74 in both Korea and Iran [26,27].

Up until now, a couple of economic evaluations have been carried out in China; however, the optimal strategy still remains uncertain [28,29,30,31,32]. Several studies have shown that the guideline-recommended strategy, i.e., starting screening at 50 years old for those smoking ≥ 20 or 30 pack-years, was cost-effective [28,29]. Another study proved that the 2018 guideline-recommended strategy was cost-effective compared with the 2021 one, indicating that lung cancer screening had a better economic performance in individuals with a minimum cumulative smoking exposure of 20 pack-years than 30 pack-years [33]. However, both these studies only probed into the starting age of 50 without mentioning another starting age, which may be the candidate for the optimal strategy. Although, another study explored different starting ages but did not take other risk factors into consideration [28,29]. Meanwhile, given that these guidelines and studies in China only investigated the stopping age of no more than 74, whereas both USPSTF and the ACR recommend annual screening until the age of 80, the appropriate age to stop screening should also be further investigated [13,14]. Moreover, only the 2018 and 2021 guideline criteria for high-risk populations were analyzed in the previous study without taking the 2022 guideline criteria into evaluation. In addition, even the most recent study in China explored optimal risk factor-based strategies including starting and stopping screening age and smoking history. The strategies with different initial screening ages were compared to their corresponding no-screening strategies using different cohorts; thus, screening strategies could not be compared directly in the same cost-effectiveness plane, making the results not comparable [30,31]. Lastly, due to significant discrepancies in economic and epidemiological statuses among Chinese regions, the optimal strategy may also vary in different regions. Therefore, in the present study, we developed a Markov model with the aim of examining the cost-effectiveness of multiple potential screening strategies and high-risk population inclusion criteria of current smokers for lung cancer screening by LDCT in a more comprehensive way in China.

## 2. Methods

### 2.1. Model Structure

**Figure 1:**
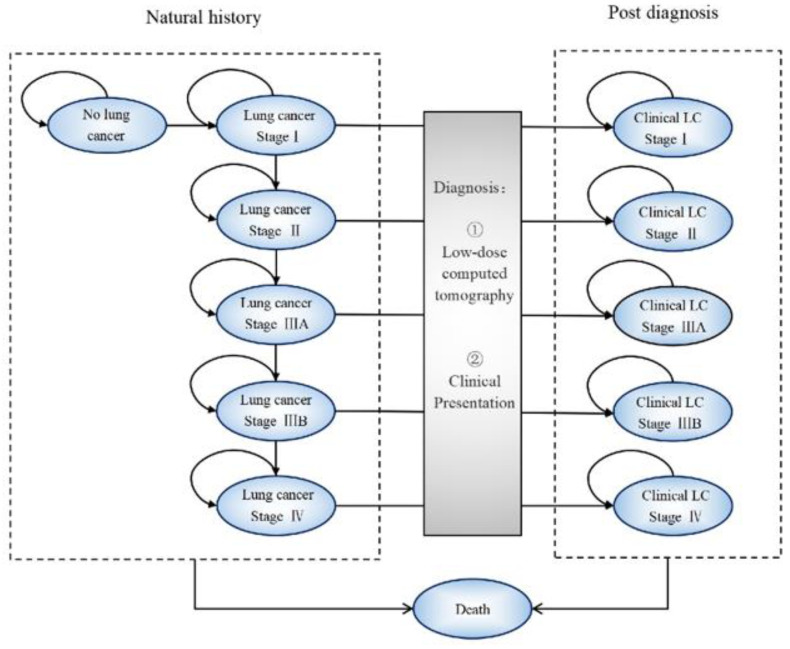
Markov model for natural history and post-diagnosis. LC, lung cancer.

A previously validated Markov model consisting of the natural history and post-diagnosis states was developed using TreeAge software to estimate the cost-effectiveness of LDCT screening [24,33]. The natural history consisted of seven states, including no apparent lung cancer, lung cancer stage I to IV and death, which represent the potential transitions for lung cancer. Healthy people could transition to stage I or remain in the no lung cancer stage. Individuals who developed lung cancer could (a) remain in the current stage; (b) progress to a higher stage; (c) get diagnosed by clinical symptoms; or (d) die of lung cancer or other causes. Since the cycle length of the Markov model was three months, those with lung cancer but still asymptomatic could also be diagnosed by LDCT screening every 4 cycles. The natural history of lung cancer development was simulated as described by previous studies in China [28,29,30,31,34]. Because the progression of lung cancer was not modeled explicitly after diagnosis, the average costs and outcomes were estimated according to the stage at diagnosis. The Markov model is provided in Figure 1.

### 2.2. Screening Strategies

Our study aimed to predict the cost and quality-adjusted life years (QALYs) of LDCT screening for current smokers combining different key characteristics of screening strategies, including screening starting and stopping ages along with smoking exposure leading to eligibility for screening. As annual screening proved to be cost-effective and recommended in most guidelines, we only evaluated the annual screening policy instead of taking other screening frequencies into consideration. The cohorts born between 1976 and 1980 (aged 40–44 years in 2020) were investigated until the age of 85 or death. Individuals entered the model at 40 years of age and then started annual screening at 40, 45, 50, 55, 60 or 65 years. The ages to stop screening were 69, 74 or 79 years. The smoking criteria based on the cumulative number of pack-years adopted by China’s national lung cancer screening guideline was also incorporated in our analysis. Finally, 36 screening scenarios were included and the scenario characteristics are shown in Table 1. The 2018 China guideline-recommended strategy (50–74 years, annual, 20 pack-years), the 2021 China guideline-recommended strategy (50–74 years, annual, 30 pack-years) and the 2022 China guideline-recommended strategy (45–74 years, annual, 20 pack-years) were included [17,18,19].

Data about screening were mainly obtained from a community-based mass screening project with LDCT for lung cancer in Guangzhou, which included 11,708 participants that were screened from 2015 to 2021 [35]. The eligible subjects were 40 to 74 years old, and those diagnosed with lung cancer within the past 5 years were excluded. Prior to the first cycle, the baseline state distribution was obtained from the first screening round of the project.

### 2.3. Clinical and Epidemiological Data

The size of the target population for different screening strategies were estimated from the smoking condition of people older than 40 years of age in the Asian population (Table 2) [36,37]. The age-specific lung cancer incidence for current smokers was determined by the incidence rate of the overall population in China and the relative risk (RR) of heavy smokes to non-smokers [4,37]. The average age-specific all-cause mortality by sex was taken from China’s population census yearbook [38]. Both of them allowed varying across age. After diagnosis, the lung cancer-specific mortality was directly dependent on the clinical stage at diagnosis. The two-year survival rates related to the stage at diagnosis based on clinically staged I–IV patients according to the tumor, node and metastasis (TNM) classification obtained from the International Association for the Study of Lung Cancer (IASLC) lung cancer staging project as well as the Chongqing cancer registration system, which included 16188 lung cancer patients from 2001 to 2018 in China, were used to calculate the transition probability after diagnosis [39,40]. Moreover, the cycle length of the Markov model was 3 months, so the transition probability should be present as a 3-month probability. Because there were different follow-up periods in various data sources, the calculation was carried out as follows if the follow-up periods were one year:

(1) $$\mathrm{Risk}\;\mathrm{for}\;\mathrm{an}\;\mathrm{event}\;\mathrm{three}\;\mathrm{months}=1-{\left(1-\mathrm{Risk}\;\mathrm{for}\;\mathrm{an}\;\mathrm{event}\;\mathrm{one}\;\mathrm{year}\right)}^{\frac{1}{4}}$$

Finally, all rates were then converted into probability.

### 2.4. Screening Parameters and Effectiveness

The screening process and nodule management were conducted according to the 2023 version of the China National Lung Cancer Screening Guideline by the National Health Commission [41]. Compared with the 2018 version, the follow-up interval for positive nodules was extended from 3 months to 6 months, which was consistent with that used in our model. For baseline screening, those with solid or part-solid nodules 5 to 15 mm or non-solid nodules 8 to 15 mm were placed on early recall. Those with nodules ≥ 15 mm or airway disease were referred immediately to pulmonologists. For annual screening, those who had new calcified nodules were placed on early recall and those with the enlargements of the original nodules, increment in solid components or airway lesions were referred immediately to pulmonologists. Early recall rates and immediate referral rates that were used to estimate the cost of the screening program as declared in the subsequent section were also obtained from a community-based mass screening project in Guangzhou including 11,708 participants in different age groups [35].

Sensitivity (probability of positive diagnosis if diseased) and specificity (probability of negative diagnosis if not diseased) values were obtained from a study that enrolled 9522 person-times over five screening rounds from 2014 to 2018 in Sichuan, China [42]. The probability that was diagnosed through LDCT screening was calculated based on the sensitivity. The proportion of the population who encountered a false-positive was estimated according to the specificity. Perfect attendance to screening was assumed for base-case analysis. Overdiagnosis, the major concern of lung cancer screenings, was considered in the scenario analysis.

### 2.5. Cost Input (Screening, Diagnosis and Treatment)

The costs considered include the cost of LDCT screening, the cost to further work up positive screening results (cost of diagnosis) and the treatment cost of lung cancer based on the stage at diagnosis. The average cost of LDCT screening is USD53.94 per time obtained from the local hospital [43]. The cost to further work up a positive result is USD333.89 including lung biopsy (USD221.14) and pre-diagnosis (USD112.75) for those who were referred to pulmonologists immediately or after a second CT scan. The cost of baseline screening and annual screening were calculated separately.

The cost of diagnosis as mentioned above and the treatment cost of lung cancer based on the stage at diagnosis were taken from other literature, which were derived from a database of a local medical insurance bureau, including 4947 patients and 107,428 relevant records; the cost of maintenance accounted for 10% of the total treatment [30,31]. All costs were adjusted to 2021 and converted into US dollars at the exchange rate of 2021 shown in the National Bureau of Statistics of China (1 US dollar = 6.4515 Chinese yuan renminbi).

### 2.6. Quality of Life

Utility for those without clinical lung cancer was extracted from studies evaluating the quality of life of the general population in China [44,45]. Because there were seldom studies that evaluated the health utility values of lung cancer patients in the Chinese mainland, the utility values of lung cancer in different clinical stages were obtained from Taiwan province by considering population characteristics [32]. Although lung cancer screening itself did not adversely affect the quality of life, disutility associated with a false-positive screening (which lasted for 3 months) was taken into account for the reason that those with false-positive results would either be recalled early or undergo unnecessary lung biopsy resulting in mental anguish [33,46]. A summary of key input parameters is presented in Table 2.

**Table 2:** Key parameter values for the Markov model.

Parameter	Baseline	Minimum	Maximum	Distribution	Reference
**Smoking prevalence in in the Chinese population**					
Male	74.1%	71.8%	76.0%	Beta	[36]
Female	5.40%	4.00%	7.00%	Beta	[36]
**Proportion of smoking amount in smokers**					
20–29 pack-years in male	30.36%	24.29% *	36.43% *	Beta	[37]
20–29 pack-years in female	10.87%	8.70% *	13.04% *	Beta	[37]
≥30 pack-years in male	34.68%	27.74% *	41.62% *	Beta	[37]
≥30 pack-years in female	6.51%	5.21% *	7.81% *	Beta	[37]
**Data about Transition probabilities**					
**Age-specific lung cancer incidence in China, years**					
40–44	0.0001414	—	—	—	[4]
45–49	0.0002867	—	—	—	[4]
50–54	0.0005538	—	—	—	[4]
55–59	0.0010131	—	—	—	[4]
60–64	0.0016089	—	—	—	[4]
65–69	0.0022856	—	—	—	[4]
70–74	0.0029973	—	—	—	[4]
75–79	0.0036277	—	—	—	[4]
80–84	0.0040047	—	—	—	[4]
85+	0.0032795	—	—	—	[4]
**RRfor high-risk population**					
Smoking 20–29 pack-years	2.70	2.16 *	3.24 *	Gamma	[37]
Smoking ≥ 30 pack-years	6.10	4.88 *	7.32 *	Gamma	[37]
**Progression rate, per cycle**					
No lung cancer to death	Time dependent	—	—	—	[38]
Stage I to Stage II	0.3558	—	—	Beta	[24]
Stage I to Stage IIIA	0.0328	—	—	Beta	[24]
Stage I to Stage IIIB	0.00000001	—	—	Beta	[24]
Stage I to Stage IV	0.0869	—	—	Beta	[24]
Stage I to diagnosis	0.0246	—	—	Beta	[24]
Stage I to death	0.1544	—	—	Beta	[24]
Stage II to Stage IIIA	0.2480	—	—	Beta	[24]
Stage II to Stage IIIB	0.0060	—	—	Beta	[24]
Stage II to Stage IV	0.1290	—	—	Beta	[24]
Stage II to diagnosis	0.0270	—	—	Beta	[24]
Stage II to death	0.1231	—	—	Beta	[24]
Stage IIIA to Stage IIIB	0.2246	—	—	Beta	[24]
Stage IIIA to Stage IV	0.1455	—	—	Beta	[24]
Stage IIIA to diagnosis	0.0811	—	—	Beta	[24]
Stage IIIA to death	0.1527	—	—	Beta	[24]
Stage IIIB to Stage IV	0.0336	—	—	Beta	[24]
Stage IIIB to diagnosis	0.5177	—	—	Beta	[24]
Stage IIIB to death	0.1853	—	—	Beta	[24]
Stage IV to diagnosis	0.6584	—	—	Beta	[24]
Stage IV to death	0.2978	—	—	Beta	[24]
**Fatality rate after treatment, per cycle**					
Stage I	0.0121	0.0097 *	0.0145 *	Beta	[39,40]
Stage II	0.0445	0.0356 *	0.0534 *	Beta	[39,40]
Stage IIIA	0.0720	0.0576 *	0.0864 *	Beta	[39,40]
Stage IIIB	0.1262	0.1010 *	0.1514 *	Beta	[39,40]
Stage IV	0.1986	0.1589 *	0.2383 *	Beta	[39,40]
**Screen Parameters**					
Sensitivity	0.8913	0.7696	0.9527	Beta	[42]
Specificity	0.9436	0.9388	0.9481	Beta	[42]
Baseline screening					
Early recalls (40 y)	0.1442	0.1154 *	0.1730 *	Beta	—
Immediate referrals (40 y)	0.0134	0.0107 *	0.0161 *	Beta	—
Early recalls (45 y)	0.1587	0.1270 *	0.1904 *	Beta	—
Immediate referrals (45 y)	0.0088	0.0070 *	0.0106 *	Beta	—
Early recalls (50 y)	0.1534	0.1227 *	0.1841 *	Beta	—
Immediate referrals (50 y)	0.0122	0.0098 *	0.0146 *	Beta	—
Early recalls (55 y)	0.1513	0.1210 *	0.1816 *	Beta	—
Immediate referrals (55 y)	0.0173	0.0138 *	0.0208 *	Beta	—
Early recalls (60 y)	0.1511	0.1209 *	0.1813 *	Beta	—
Immediate referrals (60 y)	0.0284	0.0227 *	0.0341 *	Beta	—
Early recalls (65 y)	0.1664	0.1331 *	0.1997 *	Beta	—
Immediate referrals (65 y)	0.0306	0.0245 *	0.0367 *	Beta	—
Annual screening					
Early recalls	0.0442	0.0354 *	0.0530 *	Beta	—
Immediate referrals	0.0237	0.0190 *	0.0284 *	Beta	—
**Utilities**					
Without clinical lung cancer	0.933	0.929	0.951	Beta	[44,45]
Stage I	0.840	0.756 *	0.924 *	Beta	[32]
Stage II	0.790	0.711 *	0.869 *	Beta	[32]
Stage III	0.790	0.711 *	0.869 *	Beta	[32]
Stage IV	0.770	0.693 *	0.847 *	Beta	[32]
Disutility (false-positive)	0.063	0.057 *	0.069 *	Beta	[33,46]
**Cost (USD)**					
LDCT screening	53.94	43.15 *	64.73 *	Gamma	[43]
Lung biopsy and diagnosis	333.89	267.11 *	400.67 *	Gamma	[30,31]
Treatment costs per cycle					
Clinical stage I	2258.33	1806.66 *	2710.00 *	Gamma	[30,31]
Clinical stage II	3739.69	2991.75 *	4487.63 *	Gamma	[30,31]
Clinical stage III	4066.14	3252.91 *	4879.37 *	Gamma	[30,31]
Clinical stage IV	5224.76	4179.81 *	6269.71 *	Gamma	[30,31]

LDCT, low-dose computed tomography; RR, relative risk; y, year-old. * The range of utilities were assumed to vary by ±10%; others varied by ±20%.

### 2.7. Analysis

A cost-effectiveness analysis using a Markov model was conducted from the Chinese healthcare system perspective with the application of half-cycle correction. All cost and quality-adjusted life years involved in the study would be discounted by 5% per year according to the China Guidelines for Pharmacoeconomic Evaluations. The incremental cost-effectiveness ratio (ICER) was calculated by dividing the difference in cost by the change in QALY. The WTP was considered to be 3 times the gross domestic product (GDP) per capita in China in 2021 (USD37,653 per QALY). LDCT screening would be considered cost-effective only if the ICER was less than the predefined WTP.

Uncertainty analysis was conducted to examine the model sensitivity to different parameters for the strategy in the efficient frontier. In one-way sensitivity analysis, input values were varied between the minimum and maximum to assess the influence on ICER. Probability sensitivity analysis (PSA) was also performed with 10,000 iterations, during which, different values were randomly selected from their distribution. Most of the parameters involved in the model can be assessed at the same time to further appraise the uncertainty, providing a more accurate reflection of real-world events. Finally, cost-effectiveness acceptability curves could be obtained that indicate the probability that LDCT would be cost-effective at different willingness-to-pay thresholds. The lower and upper values as well as the statistical distribution for each parameter are shown in Table 2.

Several scenario analyses were conducted for all 36 strategies to further investigate the composition of the efficient frontier in our study: (1) LDCT screening led to overdiagnosis. The average relative risk of a lung cancer diagnosis estimated based on NLST protocol and overdiagnosis rates obtained from NLST were 1.15 and 18.5%, respectively [33,47]. (2) The participation rate of the high-risk population was 35.6%, which was acquired from a one-off LDCT screening in a population-based cohort conducted from February 2013 to October 2018 in 12 cities of 8 provinces across China [48]. (3) Because there were major differences among the regions concerning the lung cancer incidence rates and economic conditions, the cost-effectiveness analysis was also conducted based on the perspective of regions from East China (Zhejiang), West China (Yunnan), South China (Guangdong) and North China (Heilongjiang) by applying the incidence rates in the different regions [49,50,51,52].

Our study conforms to the Consolidated Health Economic Evaluation Reporting Standards (CHEERS) for health interventions [53].

## 3. Result

### 3.1. Base-Case Analysis

**Figure 2:**
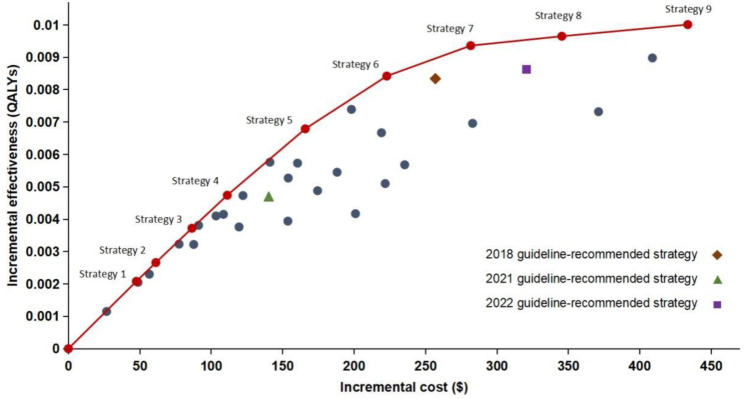
Cost-effectiveness plane of all 36 screening strategies versus no screening in the base-case analysis. QALYs, quality-adjusted life years; GDP, gross domestic product.

All screening strategies were predicted to lead to health benefits with the increase in cost. The greater QALYs as well as higher cost would be obtained when screening was started at a younger age or screening was stopped at an older age. Figure 2 shows the cost-effectiveness plane illustrating incremental costs and QALYs for each strategy. The efficient frontier, consisting of the strategies that provided the highest QALYs gained for their costs, is also presented. The 2018, 2021 and 2022 China guideline-recommended strategies were absolutely dominated by other strategies, indicating that there were other better scenarios recommended in our research. The screening strategies on the cost-effectiveness frontier and the guideline-recommended strategies are described in Table 3. Strategy 4, annual screening between ages 65 and 79 for those who smoked more than 20 pack-years, would be the best choice when the WTP threshold was two times GDP per capita (USD25,102). If the threshold was three times GDP per capita (USD37,653), strategy 6, screening for those aged 55–79 years with a 20 or more pack-year smoking history, was considered optimal. No annual screening strategy would be cost-effective if the WTP threshold was set at one time GDP per capita (USD12,551).

Smoking eligibility criteria also had a significant impact on the cost-effectiveness. According to our analysis, most of the scenarios in the efficient frontier were screening for those with a minimum cumulative smoking exposure of 20 pack-years. Meanwhile, compared with the 2021 guideline-recommended strategy with a smoking eligibility criteria of 30 pack-years, the 2018 recommendation with a smoking eligibility criteria of 20 pack-years led to an increase in QALYs of 0.00364 with higher costs.

### 3.2. Sensitivity Analysis

**Figure 3:**
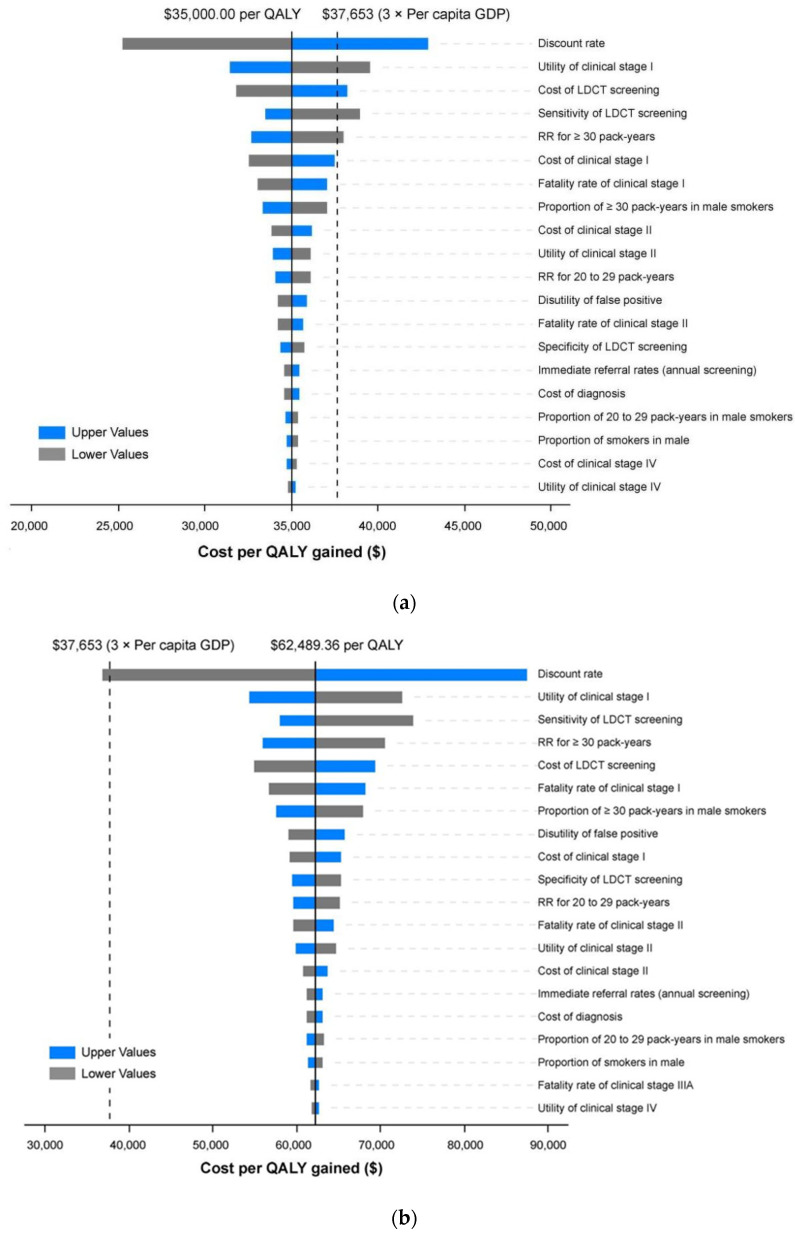
Tornado diagrams of the one-way sensitivity analysis. (**a**) Strategy 6 (55–79-year-olds with a smoking history of ≥20 pack-years) versus strategy 5 (60–79-year-olds with a smoking history of ≥20 pack-years). (**b**) Strategy 7 (50–79-year-olds with a smoking history of ≥20 pack-years) versus strategy 6 (55–79-year-olds with a smoking history of ≥20 pack-years).LDCT, low-dose computed tomography; RR, relative risk; QALY, quality-adjusted life year; GDP, gross domestic product.

Sensitivity analysis was conducted in the most optimal strategy (strategy 6, 55–79 years, ≥20 pack-years) and the promising optimal strategy (strategy 7, 50–79 years, ≥20 pack-years). The tornado diagram revealed the result of the one-way sensitivity analysis reflecting that some model parameters have a significant impact on the ICER (Figure 3). The most influential factors in the result were the discount rate, the utility of clinical stage I, the cost of LDCT screening and the sensitivity of LDCT. In contrast, the cost and utility of clinical stage IV impacted the ICER to a small degree. With the change of some parameters, such as the discount rate, the cost and utility of clinical stage I, the sensitivity of LDCT, etc., the ICER would exceed USD37,653 (three times GDP per capita in China in 2021) when applying strategy 6. For strategy 7, the ICER would also be below the threshold if the discount rate decreased.

**Figure 4:**
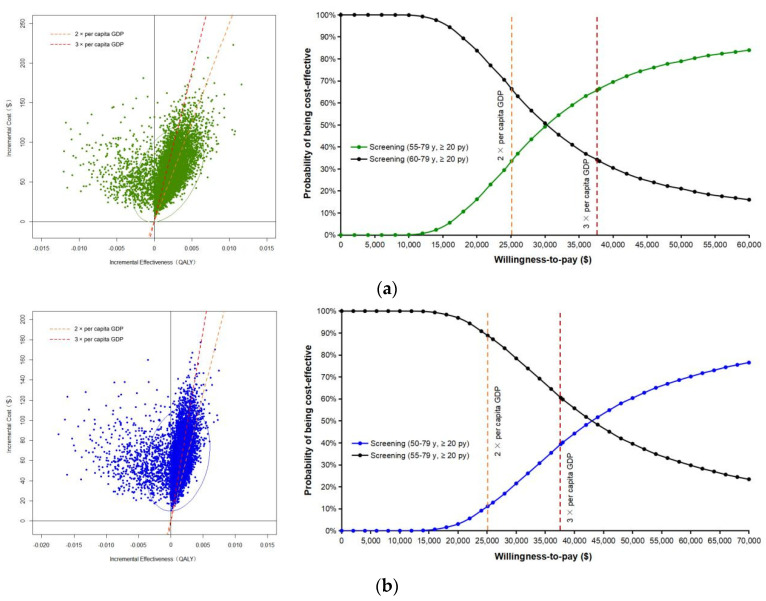
Scatter plots and cost-effectiveness acceptability curves of probability sensitivity analysis. (**a**) Strategy 6 (55–79-year-olds with a smoking history of ≥20 pack-years) versus strategy 5 (60–79-year-olds with a smoking history of ≥20 pack-years). (**b**) Strategy 7 (50–79-year-olds with a smoking history of ≥20 pack-years) versus strategy 6 (55–79-year-olds with a smoking history of ≥20 pack-years). GDP, gross domestic product; QALY, quality-adjusted life year; y, year-old; py, pack-years.

In probability sensitivity analysis, most of the scatter points fall within the northeast quadrant, indicating that LDCT usually leads to higher costs and higher QALYs in the meantime. Because the disutility conducted by false-positive results brought about higher costs and lower QALYs for those without lung cancer, some simulation points were located in the northwest quadrant in every screening strategy. The scatter plots and cost-effectiveness acceptability curves with lines indicating the WTP threshold of two and three times GDP per capita in China were displayed in Figure 4. Compared with the previous efficient scenario, strategy 6 (annual screening between ages 55 and 79 for those who smoked more than 20 pack-years) has a 65.74% likelihood, and strategy 7 (annual screening between ages 50 and 79 for those who smoked more than 20 pack-years) has a 39.45% likelihood of being cost-effective at the threshold of USD37,653.

Scenario analysis was also conducted in our study. The ICER increased when overdiagnosis, which is the major concern of lung cancer screening, was considered. In this case, it would be better to screen at 65 to 79 years of age. In addition, the ICER remained stable when incorporating lower participation rates, implying that LDCT screening was worth further extending in order to gain more survival benefits. As the cost of LDCT was one of the major impact factors of ICER according to the above one-way sensitivity analysis, lowering the LDCT price may play a vital role in the promotion of LDCT screening. In addition, starting the screening at 50 years of age may be the optimal scheme for areas with advanced economic development where lung cancer incidence showed a younger trend. For example, in Zhejiang province, compared with the preceding strategy, screening between ages 50 and 79 for persons who smoked more than 20 pack-years was recommended with the ICER of USD51,429.75, which is less than their local WTP threshold, USD52,560. On the contrary, for Yunnan province, where the incidence rate was below the national level or at a low level for the younger population, screening at 65 to 74 years of age for those with a smoking history of at least 20 pack-years was recommended when combined with the local economy situation. The optimal strategy for different WTP thresholds of each scenario is displayed in Table 4. The scatter plots and the resulting efficiency frontier of each scenario are described in detail in Figures S1–S6 and Tables S1–S6 in the Supplementary Materials.

## 4. Discussion

Our novel study explored as many as 36 potential LDCT screening strategies in China comprehensively and systematically by considering screening starting and stopping ages as well as the minimum cumulative smoking exposure leading to eligibility for screening. Meanwhile, we compared the total cost and QALYs for all potential strategies directly on the same cost-effectiveness plane, and risk factors were considered simultaneously, which was widely adopted in most international studies [33,54,55]. Previous studies in China compared the strategies with different starting ages in independent cohorts resulting in independent ICER, by which no comparison could be conducted among the strategies. According to our results, compared with no screening, all strategies lead to health benefits and higher costs for current smokers. The ICER of the strategies on the efficiency frontiers ranged from USD22,875.00 to USD244,805.56 depending on the combination of risk factors. Combined with the result of the sensitivity analysis, annual screening at 55 to 79 years of age for those who had a more than 20 pack-year history of smoking was cost-effective and gained the most clinical outcome when using a WTP threshold of USD37,653 per QALY (three times GDP per capita of China in 2021). Both the 2018, 2021 and 2022 guideline-recommended strategies were evaluated and were proved inferior to our recommended strategies based on the comprehensive scenarios in our study. In addition, targeted optimal recommendations were also provided based on local-specific economic and epidemiological statuses.

LDCT was recommended once a year starting at age 50 or 55 in many countries; however, starting screening at an earlier age, such as 45 or 50 years, was already recommended in China. This is mainly because there was a high incidence of lung cancer in people under 55 years of age in China and the incidence rate in Chinese females was far higher than in Western countries due to other risk factors such as second-hand smoke [56,57,58,59,60,61,62]. Moreover, the notable increase in Chinese GDP per capita in recent years led to a much higher WTP, especially in large cities. The above reasons resulted in a wider range of inclusion criteria for the eligible population. In our study, annual screening for those aged 50–79 with a smoking history of at least 20 pack-years was the optimal strategy in developed regions. Additionally, as WTP played an important role in drawing the conclusion, the setting of WTP became essential for decision-making. However, no research in any country has reached a final agreement. In the US, USD100,000 was the most commonly used amount in previous research [25,63,64], though some others chose USD50,000 [65]. European researchers set their WTP at around EUR30,000 to EUR60,000 [33,66]. Most Chinese researchers chose three times the GDP per capita, while researchers from Taiwan chose one time the local GDP per capita as their WTP [32]. Since different WTP thresholds resulted in different recommendations for decision-makers, whether three times the GDP per capita was rational or not still remained uncertain, especially for WTP thresholds for preventive strategies instead of curative strategies. Furthermore, once the WTP is one time the GDP per capita, annual screening might be replaced by a less frequent or even one-off screening strategy.

In addition to the strength of our study design, this research bears some other strengths in the study method. A Markov model consisting of natural history and post-diagnosis was adopted to simulate the screening program, which could implicitly account for positive biases of screening, such as length bias and lead time bias [67]. In addition, our study is based on a large community-based mass screening project with large participants and long-term follow-up, which ensures the creditability of the research to some degree [35]. Meanwhile, to provide more practical evidence to support decision-making, we considered some other probable situations in the scenario analyses including disutility due to a false-positive screening, lower participation rates and overdiagnosis. Accounting for the significant difference in economic status and cancer incidence among Chinese regions, we also chose four representative regions from the east, west, south and north of China as scenarios and finally provided diverse optimal recommendations, respectively, indicating that a diverse targeting policy should be provided regionally.

Several limitations still exist in our model. First, we have not addressed the variability in workup and associated costs that might affect the analysis. For example, studies in the US have shown significant costs associated with the workup of incidental tumor-like conditions outside the lungs, such as in the breast, thyroid and abdominal viscera. There are also increased costs for the workup of inflammatory and infectious lung nodules if strict criteria for performing the lung cancer screening CT are not enforced. Therefore, screening CT should only be performed in asymptomatic individuals after excluding recent respiratory tract infections. Similarly, the workup costs are also affected by the incidence of actionable nodules that have higher cancer probability depending on the reporting system that is adopted [68]. Second, like previous research in China, our study also only focused on current smokers [28,29,30,31]. As to former smokers and non-smokers, further investigations should be conducted on their inclusion criteria for LDCT screening. Third, due to the lack of detailed data, the sensitivity and specificity of LDCT, treatment cost and survival rate after diagnosis were assumed to be the same for people with distinct cancer stages, tumor types or locations, which was inconsistent with clinical real practice. However, this assumption was considered a small bias to the results according to clinical experts’ opinions in a previous study [24]. Fourth, the positive cut-off value of solid and partial solid nodules varied in different related guidelines (5 mm in the 2018 and 2022 guidelines, 6 mm in the 2021 guideline), and the most recognized cut-off value, 5 mm, was chosen in our study, which would influence the early recall rate, etc. However, the results were robust in the sensitivity analysis. Fifth, the probability of diagnosing other cardiopulmonary diseases such as coronary artery calcium and emphysema was not considered in our study, which may underestimate the value of screening [63,64]. Sixth, the smoking cessation benefit caused by screening was neglected in our study, which may also bring underestimation to our results [20,69]. Finally, because the screening-stopping age of the 2022 China guideline-recommended strategy from the Chinese Medical Association did not illustrate the situation perfectly, we assumed the screening-stopping age was the same as the 2019 version in our study [70].

Moreover, due to the significant heterogeneity of screening effects and cost-effectiveness, diverse and unique recommendations of the definition of high-risk individuals for LDCT screening should be offered according to the local disease incidence and economic development value instead of executing one uniform policy nationwide. Therefore, a calculator should be explored for regional decision-makers to be easily informed of an optimal strategy by inputting the local epidemiology and economic parameters. Finally, except for age, sex and smoking history, other risk factors, such as race, family history of lung cancer, body mass index, history of emphysema and so on, may also influence the incidence of lung cancer; however, these were not incorporated in our study [63]. This study has found that risk model-based strategies, using risk prediction models with varying eligibility thresholds for selecting high-risk populations for LDCT, may lead to a higher number of averted deaths, lower numbers of screenings per person and radiation-related lung cancer deaths [71]. In China, the risk prediction model for lung cancer was also conducted and may be useful to identify high-risk individuals [72]. Therefore, the cost-effectiveness of risk factor-based and risk model-based strategies is worth further investigation.

## 5. Conclusions

Our finding indicated that a number of screening strategies defined by the age to start and stop screening as well as smoking eligibility criteria may be cost-effective for current smokers from the perspective of the Chinese healthcare system. Among these, annual screening at 55 to 79 years for those with a smoking history of at least 20 pack-years is the cost-effective strategy with the best clinical effectiveness at WTP of three times the GDP per capita. However, the optimal strategy may be distinct when WTP is set at one or two times the GDP per capita. It is also reasonable to start screening earlier than 55 years of age in developed areas with high incidence under consideration of the survival benefits and costs. This is the comprehensive economic evaluation of lung cancer screening by LDCT in China, which provides a reference for the updates or revisions of the guidelines for national lung cancer massive screening in China.

## Figures and Tables

**Table 1:** Characteristics of the LDCT lung cancer screening strategies evaluated by the Markov model.

Scenario Chracteraistic	Considered Values
Age to start screening	40, 45, 50, 55, 60, 65
Age to end screening	69, 74, 79
Screening interval	Annual
Minimum cumulative smoking criteria	20 pack-years, 30 pack-years

**Table 3:** Base-case result of guideline-recommended strategies and screening strategies on the efficient frontier.

Strategy	Starting Age of Screening	Stopping Age of Screening	Cumulative Smoking Criteria	Costs (USD)	QALYs	ICER Compared to No Screening	ICER Compared to Previous Efficient Scenarios
No screening	——	——	——	552.87	15.64662	——	——
1	65	74	30 pack-years	600.45	15.64870	22,875.00	22,875.00
2	65	79	30 pack-years	614.10	15.64928	23,018.80	23,534.48
3	65	74	20 pack-years	639.36	15.65034	23,250.00	23,830.19
* 4	65	79	20 pack-years	664.10	15.65136	23,466.24	24,254.90
5	60	79	20 pack-years	718.69	15.65341	24,421.21	26,629.27
# 6	55	79	20 pack-years	775.74	15.65504	26,469.12	35,000.00
7	50	79	20 pack-years	834.48	15.65598	30,086.54	62,489.36
8	45	79	20 pack-years	898.24	15.65627	35,789.64	219,862.07
9	40	79	20 pack-years	986.37	15.65663	43,306.69	244,805.56
2018 guideline	50	74	20 pack-years	809.74	15.65496	30,799.76	Abs. dominated
2021 guideline	50	74	30 pack-years	693.13	15.65132	29,842.55	Abs. dominated
2022 guideline	45	74	20 pack-years	873.51	15.65525	37,154.11	Abs. dominated

*: Optimal strategy when the WTP threshold was 2 times GDP per capita; #: Optimal strategy when the WTP threshold was 3 times GDP per capita. QALYs, quality-adjusted life years; ICER, incremental cost-effectiveness ratio; Abs., absolutely; WTP, willingness-to-pay; GDP, gross domestic product.

**Table 4:** Result of optimal screening strategy on each scenario when the WTP threshold was 1 to 3 times GDP per capita.

Scenario	Optimal Strategy When The WTP Threshold Was 1× GDP per Capita (Screening Starting Age-Stopping Age-Smoking Criteria)	Optimal Strategy When The WTP Threshold Was 2× GDP per capita (Screening Starting Age-Stopping Age-Smoking Criteria)	Optimal Strategy When The WTP Threshold Was 3× GDP per Capita (Screening Starting Age-Stopping Age-Smoking Criteria)
**Nationwide**			
Overdiagnosis was considered	None	None	None
Participation rate was considered	None	65-79-20 pack-years	55-79-20 pack-years
**South China**	None	60-79-20 pack-years	55-79-20 pack-years
**East China**	None	60-79-20 pack-years	50-79-20 pack-years
**West China**	None	None	65-74-20 pack-years
**North China**	None	None	None

WTP, willingness-to-pay; GDP, gross domestic product. The optimal screening strategy was defined as the strategy on the efficiency frontier in which ICER was below the WTP threshold (compared to the previous efficient strategy) with the largest net benefit (most clinical outcome). GDP per capita in China: USD12,551; GDP per capita in South China (Guangdong province): USD15,234; GDP per capita in East China (Zhejiang province): USD17,520; GDP per capita in West China (Yunnan province): USD8941; GDP per capita in North China (Heilongjiang province): USD7326.

## Data Availability

The data are not publicly available due to restrictions.

## Supplementary Materials

The following supporting information can be downloaded at: https://www.mdpi.com/article/10.3390/cancers15184445/s1, Figure S1: The cost-effectiveness plane for all 36 screening strategies when the risk of overdiagnosis was considered. Table S1: Result of guideline-recommended strategies and screening strategies forming the cost-effectiveness frontier when the risk of overdiagnosis was considered. Figure S2: The cost-effectiveness plane for all 36 screening strategies when the participation rate for the high-risk population was 35.6%. Table S2: Result of guideline-recommended strategies and screening strategies forming the cost-effectiveness frontier when the participation rate for the high-risk population was 35.6%. Figure S3: The cost-effectiveness plane for all 36 screening strategies in South China (Guangdong province). Table S3: Result of guideline-recommended strategies and screening strategies forming the cost-effectiveness frontier in South China (Guangdong province). Figure S4: The cost-effectiveness plane for all 36 screening strategies in East China (Zhejiang province). Table S4: Result of guideline-recommended strategies and screening strategies forming the cost-effectiveness frontier in East China (Zhejiang province). Figure S5: The cost-effectiveness plane for all 36 screening strategies in West China (Yunnan province). Table S5: Result of guideline-recommended strategies and screening strategies forming the cost-effectiveness frontier in West China (Yunnan province). Figure S6: The cost-effectiveness plane for all 36 screening strategies in North China (Heilongjiang province). Table S6: Result of guideline-recommended strategies and screening strategies forming the cost-effectiveness frontier in North China (Heilongjiang province). Table S7: Result of all 36 screening strategies evaluated in the base-case analysis. Figure S7: Screening flow chart.
