# Supplementary material for: Cost-Effectiveness Analysis of Risk Factor-Based Lung Cancer Screening Program by Low-Dose Computer Tomography in Current Smokers in China
Source: Cancers (Basel). 2023 Sep 6;15(18):4445. doi: 10.3390/cancers15184445 (PMC10527380; doi:10.3390/cancers15184445)
Supplement: Supplementary file 1 [file cancers-15-04445-s001.zip › cancers-2517397-supplementary.pdf]

Supplementary Materials

Figure S1. The cost-effectiveness plane for all 36 screening strategies when the risk of overdiagnosis was considered. QALYs, quality-adjusted life years; GDP, gross domestic product.

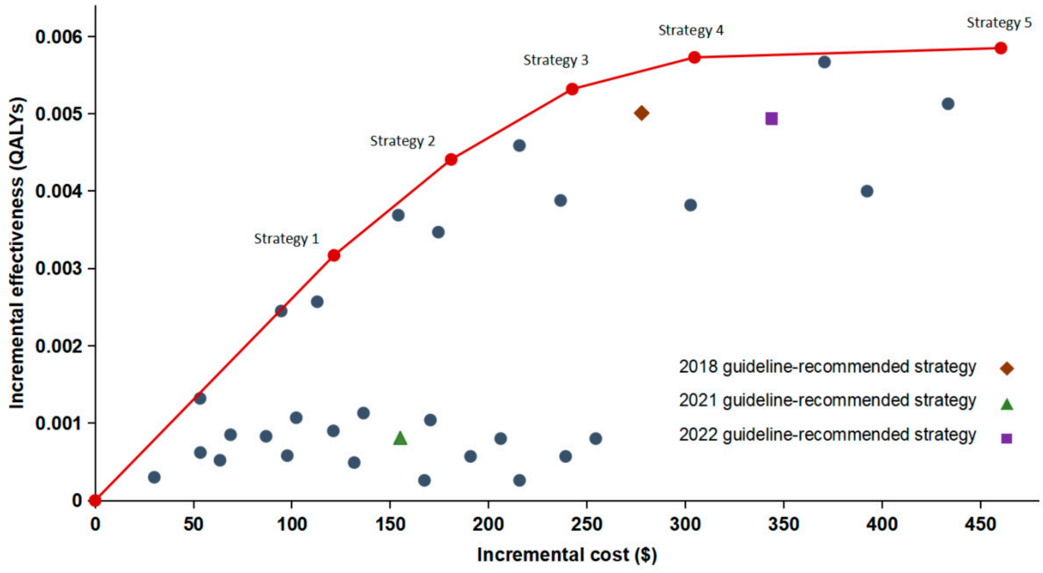

Table S1. Result of guideline-recommended strategies and screening strategies forming the cost-effectiveness frontier when the risk of overdiagnosis was considered

Strategy	Starting age of screening	Stopping age of screening	Cumulative smoking criteria	Costs ($)	QALYs	ICER compared to no screening	ICER compared to previous efficient scenarios
No screening	——	——	——	552.87	15.64662	——	——
1	65	79	20 pack-years	674.27	15.64979	38,296.53	38,296.53
2	60	79	20 pack-years	733.79	15.65103	41,024.94	48,000.00
3	55	79	20 pack-years	795.33	15.65194	45,575.19	67,626.37
4	50	79	20 pack-years	857.45	15.65235	53,155.32	151,512.20
5	40	79	20 pack-years	1013.17	15.65247	78,683.76	1,297,666.67
2018 guideline	50	74	20 pack-years	830.61	15.65163	55,437.13	Abs. dominated
2021 guideline	50	74	30 pack-years	707.93	15.64743	191,432.10	Abs. dominated
2022 guideline	45	74	20 pack-years	896.66	15.65156	69,593.12	Abs. dominated

QALYs, quality-adjusted life years; ICER, incremental cost-effectiveness ratio; Abs.: Absolutely.

Figure S2. The cost-effectiveness plane for all 36 screening strategies when participation rate for high-risk population was 35.6%. QALYs, quality-adjusted life years; GDP, gross domestic product.

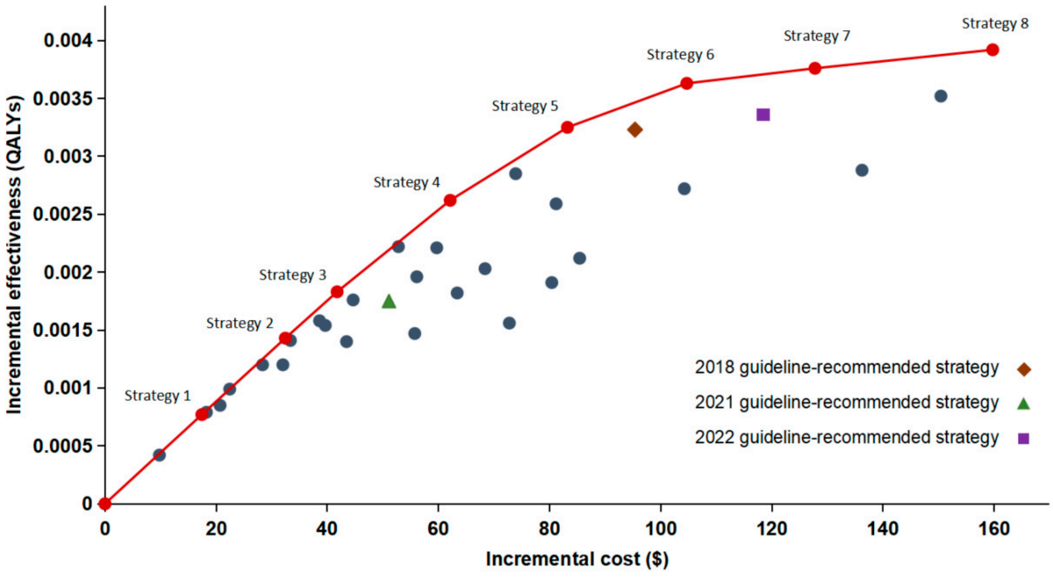

Table S2. Result of guideline-recommended strategies and screening strategies forming the cost-effectiveness frontier when participation rate for high-risk population was 35.6%

Strategy	Starting age of screening	Stopping age of screening	Cumulative smoking criteria	Costs ($)	QALYs	ICER compared to no screening	ICER compared to previous efficient scenarios
No screening	——	——	——	552.87	15.64662	——	——
1	65	74	30 pack-years	570.30	15.64739	22,636.36	22,636.36
2	65	74	20 pack-years	585.32	15.64805	22,692.31	22,757.58
3	65	79	20 pack-years	594.64	15.64845	22,825.14	23,300.00
4	60	79	20 pack-years	614.99	15.64924	23,709.92	25,759.49
5	55	79	20 pack-years	636.09	15.64987	25,606.15	33,492.06
6	50	79	20 pack-years	657.57	15.65025	28,842.98	56,526.32
7	45	79	20 pack-years	680.64	15.65038	33,981.38	177,461.54
8	40	79	20 pack-years	712.62	15.65054	40,752.55	199,875.00
2018 guideline	50	74	20 pack-years	648.25	15.64985	29,529.41	Abs. dominated
2021 guideline	50	74	30 pack-years	603.97	15.64837	29,200.00	Abs. dominated
2022 guideline	45	74	20 pack-years	671.32	15.64998	35,252.98	Abs. dominated

QALYs, quality-adjusted life years; ICER, incremental cost-effectiveness ratio; Abs.: Absolutely.

Figure S3. The cost-effectiveness plane for all 36 screening strategies in South China (Guangdong province). QALYs, quality-adjusted life years; GDP, gross domestic product.

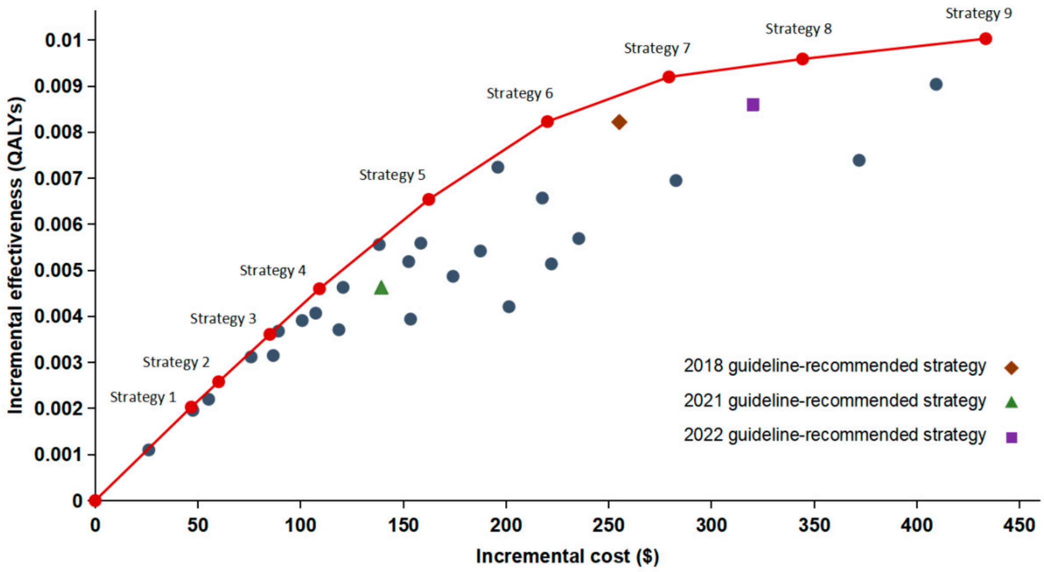

Table S3. Result of guideline-recommended strategies and screening strategies forming the cost-effectiveness frontier in South China (Guangdong province)

Strategy	Starting age of screening	Stopping age of screening	Cumulative smoking criteria	Costs ($)	QALYs	ICER compared to no screening	ICER compared to previous efficient scenarios
No screening	——	——	——	547.72	15.64606	——	——
1	65	74	20 pack-years	594.48	15.64809	23,034.48	23,034.48
2	65	79	30 pack-years	607.79	15.64864	23,282.95	24,200.00
3	65	74	20 pack-years	632.75	15.64967	23,554.02	24,233.01
4	65	79	20 pack-years	656.88	15.65066	23,730.43	24,373.74
5	60	79	20 pack-years	710.10	15.6526	24,828.75	27,432.99
6	55	79	20 pack-years	767.84	15.65429	26,746.05	34,165.68
7	50	79	20 pack-years	826.99	15.65526	30,355.43	60,979.38
8	45	79	20 pack-years	892.04	15.65565	35,904.07	166,794.87
9	40	79	20 pack-years	981.21	15.65609	43,219.34	202,659.09
2018 guideline	50	74	20 pack-years	802.85	15.65428	31,037.71	Abs. dominated
2021 guideline	50	74	30 pack-years	687.00	15.65069	30,082.07	Abs. dominated
2022 guideline	45	74	20 pack-years	867.91	15.65466	37,231.40	Abs. dominated

QALYs, quality-adjusted life years; ICER, incremental cost-effectiveness ratio; Abs.: Absolutely.

Figure S4. The cost-effectiveness plane for all 36 screening strategies in East China (Zhejiang province). QALYs, quality-adjusted life years; GDP, gross domestic product.

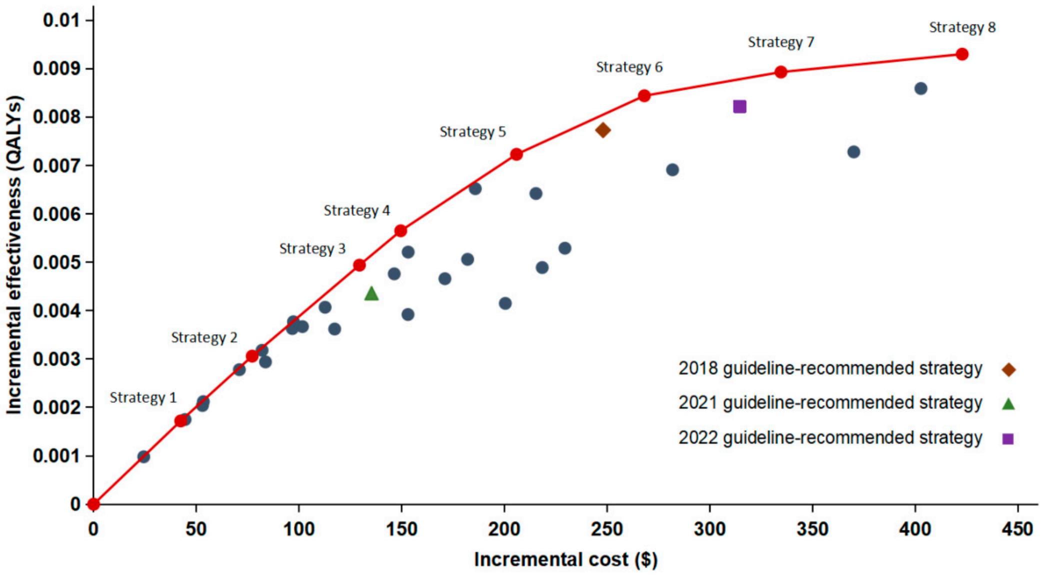

Table S4. Result of guideline-recommended strategies and screening strategies forming the cost-effectiveness frontier in East China (Zhejiang province)

Strategy	Starting age of screening	Stopping age of screening	Cumulative smoking criteria	Costs ($)	QALYs	ICER compared to no screening	ICER compared to previous efficient scenarios
No screening	——	——	——	516.31	15.65826	——	——
1	65	74	30 pack-years	558.68	15.65998	24,633.72	24,633.72
2	65	74	20 pack-years	593.50	15.66132	25,225.49	25,985.07
3	60	74	20 pack-years	645.74	15.66320	26,200.40	27,787.23
4	60	79	20 pack-years	665.84	15.66391	26,465.49	28,309.86
5	55	79	20 pack-years	722.24	15.66549	28,482.71	35,696.20
6	50	79	20 pack-years	784.47	15.66670	31,772.51	51,429.75
7	45	79	20 pack-years	851.00	15.66719	37,479.28	135,775.51
8	40	79	20 pack-years	939.25	15.66756	45,477.42	238,513.51
2018 guideline	50	74	20 pack-years	764.37	15.66599	32,090.56	Ext. dominated
2021 guideline	50	74	30 pack-years	651.63	15.66262	31,036.70	Abs. dominated
2022 guideline	45	74	20 pack-years	830.90	15.66648	38,271.29	Abs. dominated

QALYs, quality-adjusted life years; ICER, incremental cost-effectiveness ratio; Ext.:Extended; Abs.: Absolutely.

Figure S5. The cost-effectiveness plane for all 36 screening strategies in West China (Yunnan province). QALYs, quality-adjusted life years; GDP, gross domestic product.

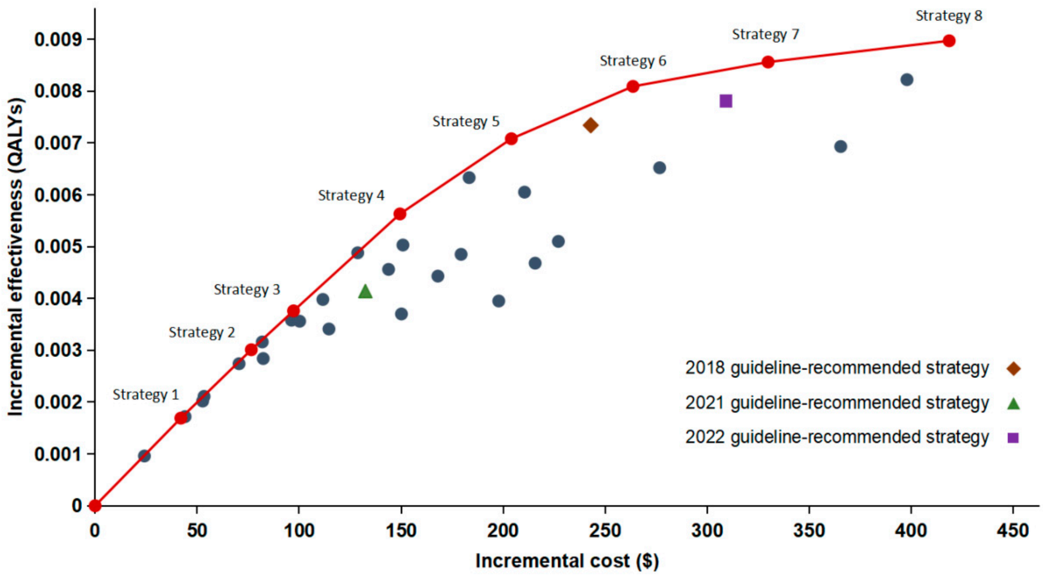

Table S5. Result of guideline-recommended strategies and screening strategies forming the cost-effectiveness frontier in West China (Yunnan province)

Strategy	Starting age of screening	Stopping age of screening	Cumulative smoking criteria	Costs ($)	QALYs	ICER compared to no screening	ICER compared to previous efficient scenarios
No screening	——	——	——	506.20	15.66554	——	——
1	65	74	30 pack-years	548.21	15.66723	24,857.99	24,857.99
2	65	74	20 pack-years	582.76	15.66855	25,435.22	26,174.24
3	65	79	20 pack-years	603.46	15.66930	25,867.02	27,600.00
4	60	79	20 pack-years	655.62	15.67117	26,539.96	27,893.05
5	55	79	20 pack-years	710.23	15.67262	28,817.80	37,662.07
6	50	79	20 pack-years	769.85	15.67363	32,589.62	59,029.70
7	45	79	20 pack-years	836.08	15.67410	38,537.38	140,914.89
8	40	79	20 pack-years	924.86	15.67451	46,673.36	216,536.59
2018 guideline	50	74	20 pack-years	749.15	15.67288	33,099.46	Ext. dominated
2021 guideline	50	74	30 pack-years	638.66	15.66968	31,995.17	Abs. dominated
2022 guideline	45	74	20 pack-years	815.38	15.67335	39,587.71	Abs. dominated

QALYs, quality-adjusted life years; ICER, incremental cost-effectiveness ratio; Ext.:Extended; Abs.: Absolutely.

Figure S6. The cost-effectiveness plane for all 36 screening strategies in North China (Heilongjiang province). QALYs, quality-adjusted life years; GDP, gross domestic product.

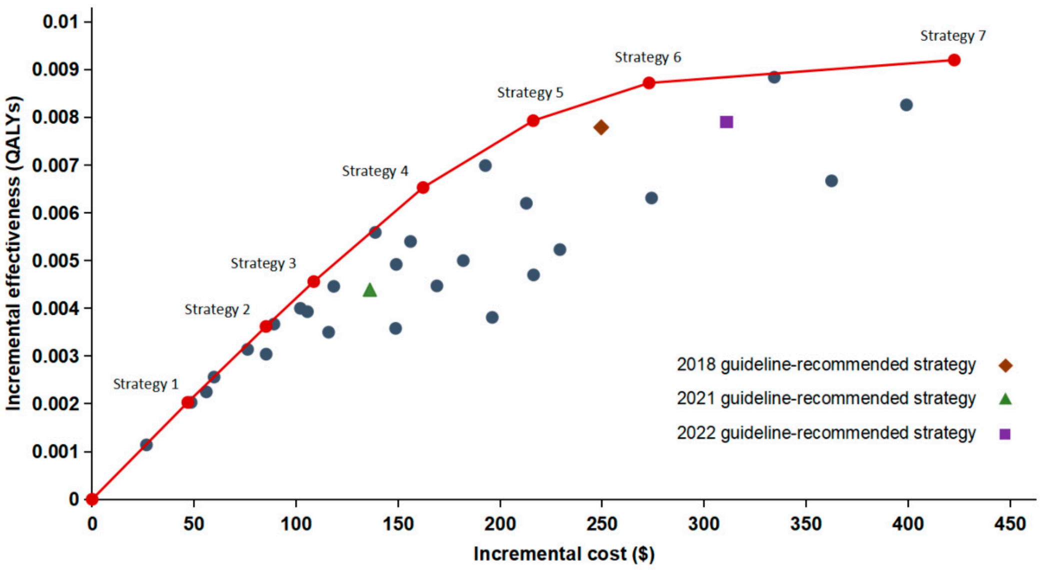

Table S6. Result of guideline-recommended strategies and screening strategies forming the cost-effectiveness frontier in North China (Heilongjiang province)

QALYs, quality-adjusted life years; ICER, incremental cost-effectiveness ratio; Abs.: Absolutely.

Strategy	Starting age of screening	Stopping age of screening	Cumulative smoking criteria	Costs ($)	QALYs	ICER compared to no screening	ICER compared to previous efficient scenarios
No screening	——	——	——	527.09	15.66341	——	——
1	65	74	30 pack-years	573.95	15.66544	23,083.74	23,083.74
2	65	74	20 pack-years	612.31	15.66703	23,541.44	24,125.79
3	65	79	20 pack-years	635.74	15.66797	23,826.75	24,925.53
4	60	79	20 pack-years	689.32	15.66994	24,843.80	27,197.97
5	55	79	20 pack-years	743.30	15.67134	27,264.82	38,557.14
6	50	79	20 pack-years	800.08	15.67213	31,306.19	71,873.42
7	40	79	20 pack-years	949.71	15.67261	45,936.96	311,729.17
2018 guideline	50	74	20 pack-years	776.64	15.67120	32,034.66	Abs. dominated
2021 guideline	50	74	30 pack-years	663.24	15.66780	31,013.67	Abs. dominated
2022 guideline	45	74	20 pack-years	838.05	15.67131	39,362.03	Abs. dominated

Table S7. Result of all 36 screening strategies evaluated in base-case analysis. QALYs, quality-adjusted life years; Ext., Extended; Abs., Absolutely.

**Screening strategy**<br>**Starting age-Stopping age-Cumulative smoking criteria**	**Costs ($)**	**QALYs**	**Category**
No screening	552.87	15.64662	Undominated
65-69 years old (30 pack-years)	579.65	15.64777	Ext. dominated
65-74 years old (30 pack-years)	600.45	15.64870	Undominated
65-69 years old (20 pack-years)	601.64	15.64867	Abs. dominated
60-69 years old (30 pack-years)	609.55	15.64892	Ext. dominated
65-79 years old (30 pack-years)	614.10	15.64928	Undominated
60-74 years old (30 pack-years)	630.35	15.64985	Ext. dominated
65-74 years old (20 pack-years)	639.36	15.65034	Undominated
55-69 years old (30 pack-years)	640.61	15.64984	Abs. dominated
60-79 years old (30 pack-years)	644.00	15.65043	Ext. dominated
60-69 years old (20 pack-years)	656.23	15.65072	Ext. dominated
55-74 years old (30 pack-years)	661.41	15.65077	Ext. dominated
65-79 years old (20 pack-years)	664.10	15.65136	Undominated
50-69 years old (30 pack-years)	672.33	15.65038	Abs. dominated
55-79 years old (30 pack-years)	675.06	15.65135	Abs. dominated
50-74 years old (30 pack-years)	693.13	15.65132	Abs. dominated
60-74 years old (20 pack-years)	693.95	15.65238	Ext. dominated
45-69 years old (30 pack-years)	706.50	15.65056	Abs. dominated
50-79 years old (30 pack-years)	706.78	15.65189	Abs. dominated
55-69 years old (20 pack-years)	713.28	15.65235	Abs. dominated
60-79 years old (20 pack-years)	718.69	15.65341	Undominated
45-74 years old (30 pack-years)	727.30	15.65150	Abs. dominated
45-79 years old (30 pack-years)	740.95	15.65207	Abs. dominated
55-74 years old (20 pack-years)	751.00	15.65401	Ext. dominated
40-69 years old (30 pack-years)	753.82	15.65079	Abs. dominated
50-69 years old (20 pack-years)	772.02	15.65329	Abs. dominated
40-74 years old (30 pack-years)	774.62	15.65172	Abs. dominated
55-79 years old (20 pack-years)	775.74	15.65504	Undominated
40-79 years old (30 pack-years)	788.27	15.65230	Abs. dominated
50-74 years old (20 pack-years)	809.74	15.65496	Abs. dominated
50-79 years old (20 pack-years)	834.48	15.65598	Undominated
45-69 years old (20 pack-years)	835.79	15.65358	Abs. dominated
45-74 years old (20 pack-years)	873.51	15.65525	Abs. dominated
45-79 years old (20 pack-years)	898.24	15.65627	Undominated
40-69 years old (20 pack-years)	923.91	15.65394	Abs. dominated
40-74 years old (20 pack-years)	961.63	15.65560	Abs. dominated
40-79 years old (20 pack-years)	986.37	15.65663	Undominated

Figure S7. Screening flow chart (a. baseline screening; b. annual screening)

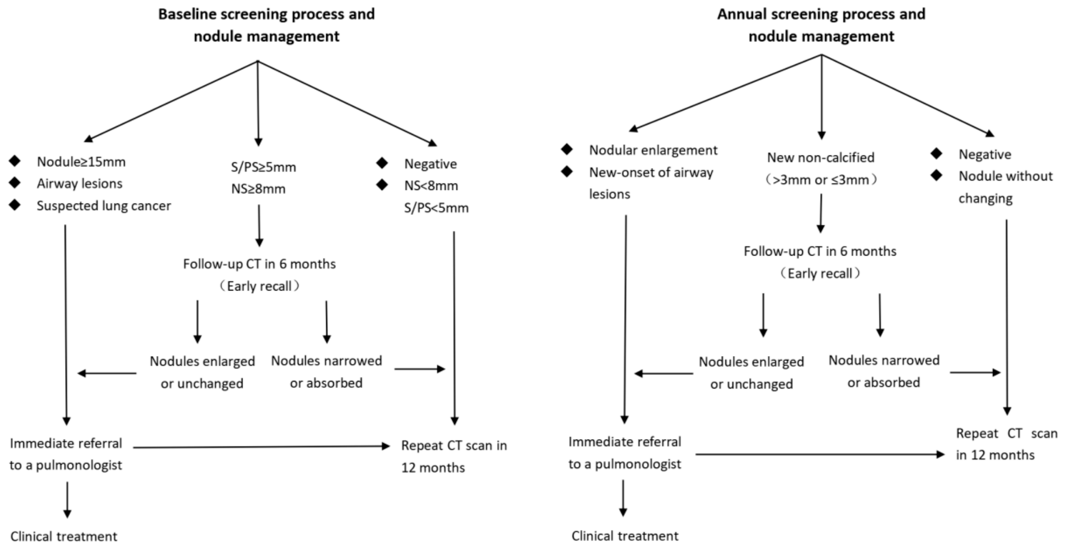

a b

*NS: Nonsolid nodules; S: Solid nodules; PS: Partial solid nodules.
